# 1155. Covering Your Tracts? The Effect of Antibiotic Prophylaxis on Respiratory Tract Infections in Pediatric Acute Lymphoblastic Leukemia

**DOI:** 10.1093/ofid/ofab466.1348

**Published:** 2021-12-04

**Authors:** Rachel Strength, Shane Cross, Ching-Hon Pui, Sima Jeha, Ashleigh Gowen, Joshua Wolf, Joshua Wolf

**Affiliations:** 1 University of Tennessee Health Science Center, Memphis, Tennessee; 2 St. Judge Children’s Research Hospital, Memphis, Tennessee; 3 St. Jude Children’s Research Hospital, Memphis, Tennessee; 4 St. Jude’s Children’s Research Hospital, Memphis, TN

## Abstract

**Background:**

Antibiotic prophylaxis decreases rates of febrile neutropenia and systemic infection in children with acute lymphoblastic leukemia (ALL). However, it is unknown whether prophylaxis prevents or ameliorates the severity of specific types of infections like upper respiratory tract infections (URTI) or lower respiratory tract infections (LRTI).

**Methods:**

This is a retrospective, observational convenience cohort study of children with newly-diagnosed ALL, comparing respiratory tract infections (RTI) in participants receiving no antibiotic prophylaxis, levofloxacin prophylaxis, or non-levofloxacin prophylaxis. Information regarding the presence of URTI or LRTI, identified respiratory viruses, hospitalization, oxygen supplementation, and ICU admission was collected through medical record review. The proportion of participants in each group was estimated and compared between groups using Fisher’s exact test and the Kruskal-Wallis test.

**Results:**

Of 262 evaluable participants, 126 received no antibiotic prophylaxis, 59 received levofloxacin prophylaxis, and 77 received non-levofloxacin prophylaxis, with a total of 136 children getting any antibiotic prophylaxis regimen. In the no-prophylaxis group, 22/126 (17.4%) had RTI, compared to 23/136 (16.9%) in the prophylaxis group. There was no significant difference in the numbers of LRTI and URTI, with or without an identified respiratory virus, regardless of the presence or type of antibiotic prophylaxis. Participants receiving prophylaxis did not have a significantly different risk of hospitalization, oxygen supplementation, or ICU admission.

Participant Characteristics

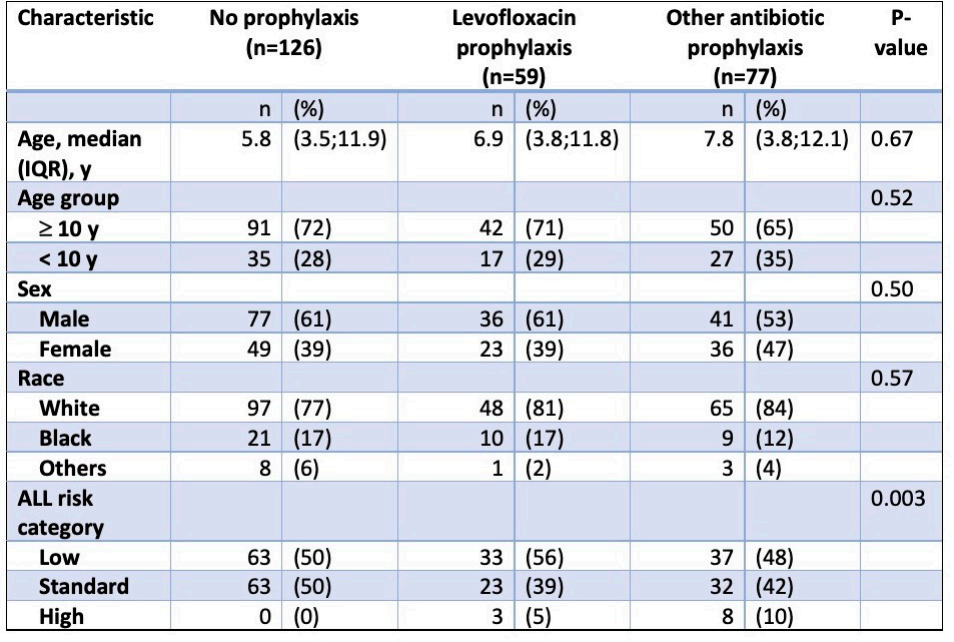

Comparisons of levofloxacin prophylaxis, other prophylaxis, any prophylaxis, and no prophylaxis

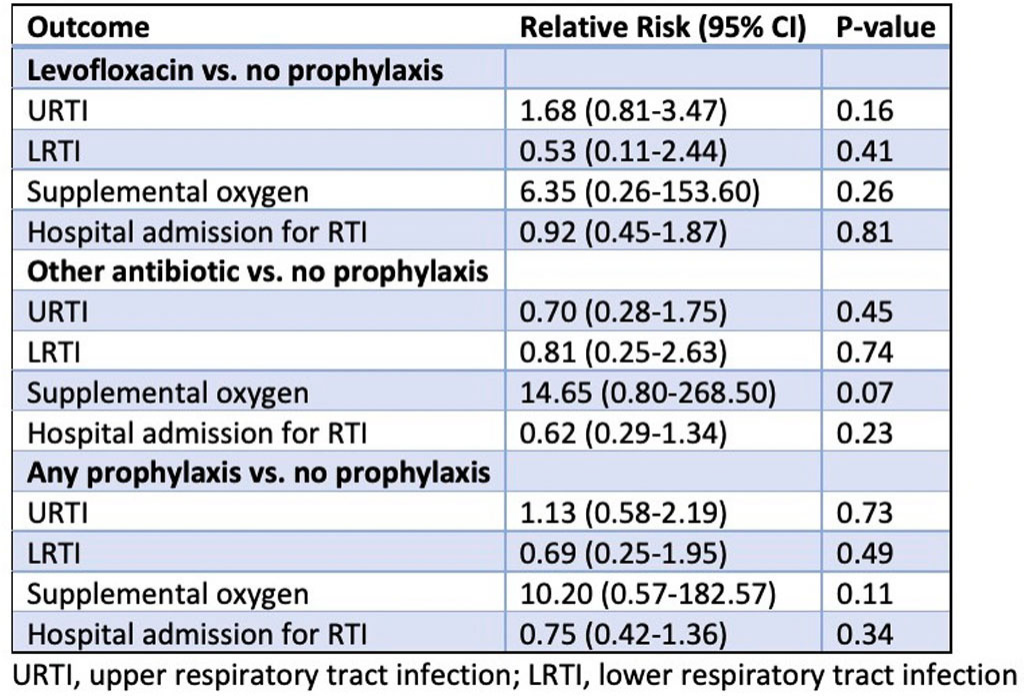

**Conclusion:**

There was no observed difference in RTI, hospitalization, oxygen supplementation, or ICU admission for RTI between participants receiving or not receiving antibiotic prophylaxis in this cohort. Because of the relatively low number and severity of respiratory infections, and the high proportion that are viral in etiology, it would likely take a very large sample size to determine the impact of antibacterial prophylaxis on respiratory infections during induction therapy for pediatric ALL.

**Disclosures:**

**Joshua Wolf, MBBS, PhD, FRACP**, **Karius Inc.** (Research Grant or Support) **Joshua Wolf, MBBS, PhD, FRACP**, Nothing to disclose

